# A Randomised Controlled Trial of a Play-Based Intervention to Improve the Social Play Skills of Children with Attention Deficit Hyperactivity Disorder (ADHD)

**DOI:** 10.1371/journal.pone.0160558

**Published:** 2016-08-16

**Authors:** Sarah Wilkes-Gillan, Anita Bundy, Reinie Cordier, Michelle Lincoln, Yu-Wei Chen

**Affiliations:** 1 School of Allied Health, Australian Catholic University, North Sydney, NSW, Australia; 2 Faculty of Health Sciences, The University of Sydney, Sydney, NSW, Australia; 3 School of Occupational Therapy and Social Work, Curtin University, Perth, WA, Australia; Cardiff University, UNITED KINGDOM

## Abstract

There is a need for effective interventions to address the social difficulties of children with ADHD. This randomised controlled trial examined the effectiveness of a play-based intervention for improving the social play skills of children with ADHD in peer-to-peer interactions. Children with ADHD (5 to 11 years) were randomised to an intervention-first (*n* = 15) or waitlist control-first group (*n* = 14). Participants allocated to the control-first group received the intervention after a 10-week wait period. Children invited a typically-developing playmate and parents of children with ADHD participated. The intervention involved: six clinic play-sessions, weekly home-modules and a one-month home follow up. The Test of Playfulness (ToP) was scored by a blinded rater. Parent reported treatment adherence was used to assess treatment fidelity. Between group statistics were used to compare the change of the intervention-first (10-week intervention period) and control-first (10-week wait period) groups. Once all children had received the intervention, repeated measures ANOVA, post hoc Least Significance Difference tests and Cohen’s-*d* were used to measure effect. Changes in ToP social items were analysed using Friedman’s ANOVA. Linear regression analyses were used to identify variables that predicted change. The control-first group did not change during the wait period. The change in the intervention-first group was significantly greater than the change in the control-first group (during the wait period). When the data from the two groups were combined, the mean ToP scores of the children with ADHD (*n* = 29) improved significantly following the intervention, with a large effect from pre to post intervention and from pre intervention to follow up. Children maintained treatment gains at follow up. All ToP social items improved significantly following the intervention. The findings support the use of play involving parent and peer mediated components to enhance the social play skills of children with ADHD.

***Trial Registration*:** Australian New Zealand Clinical Trials Registry ACTRN12614000973617

## Introduction

Attention deficit hyperactivity disorder (ADHD) is one of the most prevalent developmental disorders [1, 2]. The costly, long-term nature of the disorder presents a major public health challenge [3]. As a result, ADHD is widely researched; with much focus on ameliorating children’s deleterious social difficulties [4].

The social difficulties of children with ADHD are profoundly greater than those experienced by typically-developing peers [4–7]. Many children with ADHD have social difficulties [8], including aspects of social cognition [9]. Some researchers theorise that these social difficulties are attributable to deficits in skill acquisition [10].

One core area of social difficulty for children with ADHD is peer interactions, particularly within the context of play. In peer-to-peer play interactions, children with ADHD had difficulty with: sharing, supporting, responding to social cues, cooperative play, perspective-taking and were more self-focused when negotiating compared to control children [11, 12]. These findings are consistent with behavioural inhibition models describing children with ADHD as having difficulty with forethought, problem-solving and performing skills in the moment [13, 14]. Furthermore, these findings align with research into aspects of social cognitive difficulties in children with ADHD, including empathy [11, 15, 16] and theory of mind [9, 17]. In his model, Barkley [13] postulated that children with ADHD would have difficulty with the regulation of emotions. Specifically children with ADHD would demonstrate immediate emotional reactivity to emotionally charged events and fewer anticipatory emotions toward future events due to decreased capacity for forethought. Moreover, children with ADHD may view their interactional difficulties as external and outside of their control [18], suggesting that children with ADHD have a skills performance, rather than knowledge, deficit. Therefore, intervention may be more effective when children are supported to perform the skills in the naturally-occurring social environment [13] and when aspects of social cognition are targeted.

As a result of these difficulties, children with ADHD are frequently rejected by their peers and have fewer meaningful friendships [5, 19]. Further, parents of children with ADHD reported difficultly supporting their child’s friendships [20, 21], arranged fewer play-dates and were more critical of their child’s interactions than control parents [22].

Psychosocial interventions including behavioural and social skills training approaches have minimal evidence for improving the social difficulties of children with ADHD [23]; a recent systematic review reported that current approaches had limited effectiveness [24]. The social validity of traditional social skills training approaches has been questioned due the removal of children from the natural environments where they develop social skills and experience inter-personal difficulties. In social skills training approaches the peer group and adult involvement often involves rehearsal of socially acceptable social skills and structured games, where adults use behavioural consequences for desired/undesired social interactions. The delivery of treatment in group contexts where children have similar diagnoses and social difficulties has also been questioned due to placing a too greater demand on children developing new social skills [23–25]. Other explanations for the outcomes of current social skills training approaches include a lack of: 1) parent involvement, 2) inclusion of pro-social peers, 3) application of techniques across multiple contexts, 4) connection to theoretical frameworks which consider children’s underlying impairments, and 5) methodological quality [23–25].

### The Importance of Developing Complex Interventions

With the acknowledgement that non-pharmacological healthcare interventions are complex and difficult to successfully design, implement and evaluate, the United Kingdom’s Medical Research Council (UKMRC) developed guidelines to provide structure for the development of such interventions [26–28]. The UKMRC guidelines emphasise a systematic, phase-based approach to take research from the theoretical phase and pilot trials, to controlled definitive RCT trials which have the capacity to be implemented in community settings [26–28].

The play-based intervention under investigation has undergone initial stages of development. Further development of the intervention is important as interventions targeting the social impairments of children with ADHD have demonstrated minimal effectiveness [24].

### Theoretical Framework of the Play-Based Intervention

After comparing the peer-to-peer play interactions of 350 children, of which 112 had ADHD, Cordier and colleagues [29] developed a theoretical model with principles to guide the design of interventions planning to address the social difficulties of children with ADHD. Central to the model was the premise that free child-led play is the primary occupation of children and the context which facilitates children’s social development [30]. Within the model is the following definition of play, which is operationalised by the Test of Playfulness (ToP) as: “A transaction between the individual and the environment that is internally control, intrinsically motivated, free from unnecessary constraints of reality, and requires framing (i.e., giving and reading social cues) [31].” Each principle had been recommended for future psychosocial interventions for children with ADHD in previous studies [23, 25] and included: 1) *capturing children’s intrinsic motivation*, through the natural context of play, 2) *facilitating the development of interpersonal empathy*, 3) *including a regular typically-developing playmate*, for friendship development, and 4) *involving parents*, to assist skill generalisation.

### Pilot Trials of the Play-Based Intervention

Using the Cordier and colleagues [29] principles and the ToP [31], we tested a therapist-delivered intervention where 14 children with ADHD, their playmates and parents attended 7 weekly one-hour clinic sessions. During this phase, a fifth principle was added. *Therapist-modelling* was required to promote cooperative play between the dyad (children with ADHD and their playmate) and to support children with ADHD to use the target skills as natural peer interactions unfolded. One therapist provided the children with video-feedback on their previous social interactions before playing in the playroom with the dyad. The second therapist worked with the children’s parents; discussing the application of strategies at home. Children with ADHD made large, significant improvements in their social play skills from pre to post intervention [32], which were maintained 18 months following the intervention [20].

To offset the resource intensiveness of the therapist-delivered intervention and increase parent involvement, we developed a parent-delivered version of the intervention. Parents used a DVD and manual resource which contained twelve modules; each designed to address an area of social difficulty experienced by children with ADHD. Parents watched the DVD with their child at home, before facilitating a weekly play-date. Children also received three clinic sessions. The intervention was tested with five children from the therapist-delivered intervention [21], before it was tested with nine children who had not received the intervention [33]. Children made large, significant improvements following intervention which persisted to a one-month home follow up [33].

Given the long-term nature of the social difficulties experienced by children with ADHD, the lack of intervention effectiveness and the complex nature of conducting methodologically sound RCTs and large-scale community trials, further development and evaluation of the play-based intervention is essential. Conducting a definitive RCT of the play-based intervention is crucial in responding to the recommendation outlined in a recent Cochrane systematic review; the continued implementation and evaluation of rigorous RCTs aiming to improve the social skills and interactions of children with ADHD [24]. Such research is key to advancing our understanding of developing interventions that successfully address the complex needs of children with ADHD and to minimise the large gap between conducting pilot trials and implementing the intervention in costly community-based trials [26–28].

### Research Aims

The aim of this RCT was to examine the effectiveness of a play-based intervention for improving the social play skills of children with ADHD in peer-to-peer interactions. The RCT protocol, parents’ treatment adherence, and participant variables that may predict intervention change are reported with aim to elucidate the findings. The intervention design was based on the Cordier and colleagues [29] principles and combined components from the therapist- and parent-delivered interventions. We postulated that the play-based intervention with a strong theoretical model, and parent and peer involvement would yield increases in the children’s social play skills. Using the ToP [31] we tested the following hypotheses:

1. The change in the overall play skills of children with ADHD in the intervention-first group during their intervention phase will be significantly greater than the change in the overall play skills of children with ADHD in the control-first group during their control phase (10-week wait);
2. The overall play skills of all children with ADHD will improve significantly from pre to post intervention, with skills generalising to the home environment; and
3. All ToP items related to social skills will improve significantly from pre to post intervention, and generalise to the home environment.

## Methods

### Trial Design

This randomised controlled trial (RCT) was a two group parallel trial. In this single site trial, participants were randomly assigned to an intervention-first or control-first group. The intervention-first group received a 10-week play-based intervention. The control-first group received no treatment for 10-weeks after which they engaged in the 10-week play-based intervention. The Consolidated Standards of Reporting Trials (CONSORT) 2010 guidelines for evidence-based reporting of RCTs were used to report this trial [34].

### Trial Protocol

The trial protocol was registered with the Australian New Zealand Clinical Trials Registry. Given this was a psychosocial trial and not a drug trial, we were not aware that we needed to register the protocol prior to recruitment. Therefore the protocol was submitted post hoc. The authors confirm that all ongoing and related trials for this intervention are registered. This trial was approved by the University of Sydney’s Human Research Ethics Committee (approval number: 2013/109) on the 21/3/3013 prior to participant recruitment.

All informed consent procedures were approved by the ethics committee. Parents and children over 7 years provided informed written and verbal consent by completing the appropriate consent form. Due to the developing reading and writing skills of young children, children under 7 years provided verbal assent in the presence of their parents and researchers. Children provided verbal assent after the researcher had explained to the child what they would be doing as part of this research. Verbal assent was recorded/documented by the researcher writing the child’s name and date on a consent form for under 7 year olds. Parents also provided informed written and verbal consent on behalf of their child by completing the appropriate consent form. Participants were recruited from May 2013 to July 2014 and follow-up data were collected by October 2014.

### Participants

**Fig 1:**
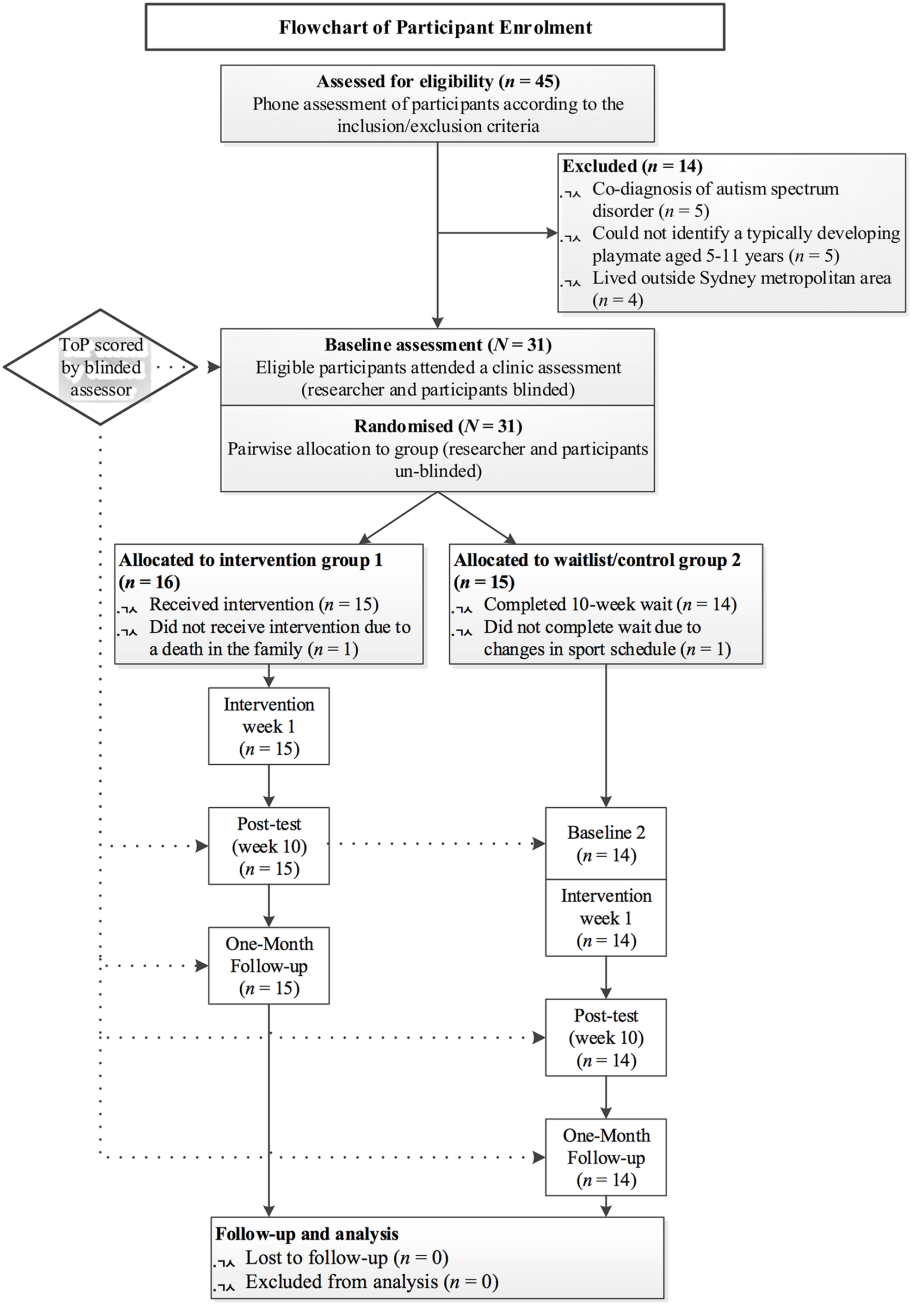
CONSORT flow diagram of the process through the phases of a parallel randomised trial of two groups, enrolment to data analysis (Moher et al., 2010).

Following ethical approval participants were recruited using convenience sampling. A recruitment flyer was distributed to paediatric services and three ADHD parent support groups across metropolitan Sydney, Australia. The flyer was also distributed across national online ADHD support groups and a media release through the University of Sydney’s website. Between April 2013 and May 2014, parents of 45 children with ADHD contacted the first author; 31 of those met the inclusion criteria. See Fig 1.

Participants were children with ADHD (*n* = 31) who each invited a known, typically-developing playmate (*n* = 31). One family enrolled three children with ADHD and two families enrolled two children with ADHD. Of these three families, two enrolled all eligible children in their family to participate in the study and one family enrolled two of their four children eligible for the study. One parent of each child with ADHD also participated. Two participants discontinued after the baseline assessment (see Fig 1). Demographic information is reported on 29 children with ADHD and their 25 parents (see Table 1).

**Table 1:** Participant Demographics.

	Intervention-First		Control-First	
**Parent Demographic Variables**^a^	*Participants*	*Playmate*	*Participants*	*Playmate*
Mean age in years (SD)	41.7 (7.0)	42.0 (4.0)	41.5 (6.0)	43.0 (4.2)
Born in Australia	8 of 12	8 of 12	10 of 13	8 of 13
Qualifications: degree or diploma	93%	93%	87%	100%
Occupation: requires tertiary qualifications	60%	47%	57%	64%
**Child Demographic Variables**	*Participants*	*Playmate*	*Participants*	*Playmate*
Mean age in years and months (SD)	8.2 (1.5)	8.5 (1.9)	8.5 (1.7)	7.9 (2.3)
Male	13 of 15	10 of 15*	12 of 14	3 of 14*
Born in Australia	14 of 15	14 of 15	12 of 14	13 of 14
**ADHD Symptomology (CCBRS)**^b^				
Hyperactivity symptoms	75^c^ (13.0)	49 (11.0)	74^c^ (12.8)	50 (7.9)
Inattention symptoms	80^c^ (11.7)	53 (10.8)	81^c^ (9.8)	50 (9.4)
Oppositional behaviour	75^c^ (13.4)	59 (14.6)	76^c^ (13.0)	52 (11.0)
Generalized anxiety disorder	71^c^ (11.5)	54 (7.8)	73^c^ (12.9)	51 (9.9)
Social problems	75^c^ (15.0)	50 (6.7)	81^c^ (13.7)	51 (11.2)
Language problems	64 (14.2)	46 (7.5)	63 (10.5)	50 (11.3)
ADHD subtypes				
Predominantly Inattentive	5 of 15	-	6 of 14	-
Predominantly Hyperactive/Impulsive	1 of 15	-	0 of 14	-
Combined Subtype	9 of 15	-	8 of 14	-
Medication taken for ADHD	9 of 15	-	11 of 14	-
Sibling as playmate	8 of 15	-	8 of 14	-
Age difference in child dyad, years/months	1.8 (1.2)	-	1.9 (1.5)	-

Notes.

^a^Some mothers enrolled more than one child in the program. Demographic information is therefore reported on 25 mothers of children with ADHD and 26 mothers of playmates.

^b^The CCBRS was used to confirm the diagnosis of ADHD.

^c^Mean scores were above the clinical cut-off, T-scores ≥ 70 on the DSM-IV subscales for children with ADHD. Playmates scored below the borderline clinical cut-off (T-scores ≤ 65) on all subscales.

*Only one differences was found between the ADHD (intervention vs. control) and playmate (intervention vs. control) groups across all interval level (i.e., CCRBS scores; *t*-tests), and nominal data variables (i.e., gender, medication use, playmate type, ADHD subtype; McNemar’s test). There were significantly more male playmates in the intervention-first group (*p* = .04).

#### Children with ADHD

To be included in the study, children with ADHD needed to be between 5 and 11 years with a formal diagnosis of ADHD made by a paediatrician or psychiatrist, using recognised diagnostic procedures such as the American Psychiatric Association’s Diagnostic and Statistical Manual 4^th^ edition (DSM-IV). The DSM-IV was used as the study commenced in April 2013. Children were included if they presented with co-morbid difficulties (i.e., language difficulties, conduct disorder) and were excluded if they were diagnosed with other major developmental disorders (i.e., intellectual disability, autism spectrum disorder). Children continued to take any medication prescribed for ADHD.

#### Typically-developing playmates

Playmates were required to be typically-developing children aged between 5 and 11 years. Playmates were included provided they did not have a diagnosis of ADHD or any other developmental disorder and no concerns had been raised by parents or teachers about the children’s development. Playmates needed to be a peer or, when the children did not have a regular playmate, a sibling who had regular weekly interactions with the child with ADHD. Playmates already known to the child with ADHD were included to promote friendship development and provide continued opportunities for social interaction.

#### Parents of children with ADHD

One parent of the children with ADHD was required to attend clinic sessions and complete home activities with their child. Two fathers and twenty-three mothers attended all intervention sessions and completed home activities with their child. For 14 of the 29 children, both parents attended the clinic sessions. Parents were asked to maintain their child’s medication status throughout the intervention and to report any changes. The therapist monitored the consistency of medication use throughout the study. Use of current medication was permitted during the testing sessions in order to obtain accurate baseline information on how the child interacted in usual circumstances.

#### Screening of ADHD symptoms for inclusion

The Conners Comprehensive Behavior Rating Scales (CCBRS) [35] were used to confirm if children with ADHD presented with symptoms consistent with ADHD upon entry to the study (i.e., scoring above the clinical cut-off, T-scores ≥ 70, on the DSM-IV subscales). Playmates required scores below the borderline clinical cut-off, T-scores ≤ 65 for DSM-IV subscales, indicating the absence of symptoms consistent with ADHD and other diagnoses [35]. As the playmates were required to model desired social skills during the intervention, parents of children with ADHD were encouraged to invite playmates who did not experience the same degree of interactional difficulties as their child (i.e., T-scores ≤ 65 for behaviour, social and communication subscales of the CCBRS). For a profile of child symptomology see Table 1.

### Instruments

#### The Test of Playfulness (ToP)

The ToP [31] was used to examine children’s play skills in peer-to-peer play interactions pre, post and one-month following the intervention. The ToP is a 29-item unidimensional instrument that requires observational scoring. The ToP is suitable for children with and without disabilities aged 6 months to 18 years [31, 36]. Each item is rated on a 4-point scale to reflect extent, intensity, or skilfulness. The ToP contains nine items that reflect social skills: 1) the skill of *initiating* interactions, 2) the skill of *negotiating*, 3) the skill of *sharing*, 4) the skill of *supporting* another, 5) the extent of time engaged in *social* interactions, 6) the intensity of involvement with another in *social* interactions, 7) the *social* skill when interacting with another, 8) the skill of *giving* verbal and non-verbal cues, and 9) the skill of *responding* to others’ verbal and non-verbal cues [33].

The ToP has evidence for excellent inter-rater reliability (data from 96% of raters fit the expectations of the Rasch model); moderate test-retest reliability (intraclass correlation 0.67 at *p* < .01) [37] and construct validity (data from 93% items and 98% of people fit Rasch expectations) [38].

#### Conners Comprehensive Behavior Rating Scales (CCBRS)

The parent-rated CCBRS [35] is a screening tool suitable to identify symptoms consistent with diagnoses and behavioural difficulties in school-aged children. The CCBRS is a reliable tool: Cronbach’s alpha .67 to .97, test—retest reliability coefficient .56 to .96 (*p* < .001), and inter-rater reliability coefficients .50 to .89 (*p* < .001). The CCBRS also has evidence of discriminative validity, with a mean classification accuracy of 78% across forms [35].

### Procedure

Prior to initiation of the study, the necessary sample size was determined. To calculate the required sample size we used G*Power software (version 3.1.9.2) using the following parameters: 1) desired power (0.8), 2) statistical test (ANOVA), 3) alpha value (.05), and 4) expected effect (> .5 large); which generated the total sample size of *n* = 30. The expected effect was based on three pilot studies [21, 32, 33]. From these studies, it was determined a recruitment period of one year was required with an expected dropout rate of 10%.

#### Concealment and randomisation

An even number of opaque envelopes containing slips of paper labelled ‘group 1, intervention’ or ‘group 2, waitlist’ were prepared and sealed by the first author. When parents enrolled in the study, a baseline assessment was scheduled over the phone. Only information regarding the inclusion criteria and the assessment date/time were collected prior to randomisation. Demographic data were collected after group allocation; thus the intervention dyads (child with ADHD and playmate) were not matched with the control dyads on any demographic or other variables. As recruitment was expected to be sporadic, randomisation was conducted with a block size of two. Within this design, simple randomisation was used to assign one of each two children who entered to each group (1:1 allocation ratio) [34, 39].

Once two parents had booked a baseline assessment, a sealed envelope from each group and the times of the baseline assessments were taken to an academic staff member not involved in the research. The person shuffled the envelopes and used a coin toss to pick one of the two times, writing it on one of the sealed envelopes. The researcher left the room while the academic staff member completed the procedure. The sealed envelopes were then placed in a client folder which was taken to the baseline assessment. To avoid potential contamination between the waitlist and intervention groups, families who enrolled more than one child with ADHD all received the same sealed group allocation envelope during the randomisation process. Moreover this was done for practical reasons to reduce participant burden, thus preventing parents having to bring their children for therapy over a prolonged period of time (20 weeks). To ensure concealment procedures, the assessors were blinded to treatment allocation for all participants, regardless of the family relationship. While the researchers knew that children in the same family would receive the same allocation, it was not revealed to them what the treatment actually was. Additionally the blinded rater was not aware of any familial relationships.

#### Enrolment and baseline assessment

During the baseline assessment, the researchers and participants were blinded to group allocation. All baseline assessments were booked on Saturdays and were scheduled for an hour at the the REMOVED FOR PEER REVIEW Research Clinic. Participating families received the intervention and onsite parking free of cost; no other reimbursement was provided. The baseline assessment involved each dyad (children with ADHD and their playmate) playing for 20-minutes in a clinic playroom. The playroom contained a one-way-mirror and wall-mounted video-camera. The playroom was consistently set up with a variety of toys including: a basket-ball hoop, bowling set, soft bat and ball games, cars, figurines, nerf guns, a tent, dress-ups, play-doh, a sand box, floor games (e.g., Twister^™^) and toys from electronic games (e.g., Angry Birds^™^, Club Penguin^™^). Each dyad played in the playroom without an adult. Children were introduced to the space and the playroom rules: have fun and come out if you need an adult. The therapist and parent observed the children from behind the one-way-mirror. During this time, a therapist-parent consultation took place where the child’s social difficulties and client history were discussed. The therapist closely observed the playmate’s interactional skills during the baseline assessment to screen for their suitability for inclusion in the program. At the end of the baseline assessment, the therapist handed the parent the sealed allocation envelope and ongoing intervention session times were scheduled.

#### Process evaluation strategies

The first author delivered the intervention. To ensure the provider’s skill in delivering the treatment, uniformity of treatment delivery, and receipt of the treatment, process evaluation strategies were employed (see Table 2) [40].

**Table 2:** Process evaluation and treatment fidelity strategies for the intervention.

Category	Goal / Description	Strategies
Monitor and improve provider training	Ensure provider skill acquisition	The first author had delivered a similar intervention protocol in three previous studies with the third author^a^. Before delivering the intervention, the first and third authors met to plan the intervention strategies. This was done using standardised protocols/checklists implemented in the pilot studies.
Monitor and improve treatment delivery	Ensure the treatments are being delivered/ adhered to in the way in which they were conceived with regard to content and treatment dose.	*Parents*. Parents received individual training in week 1 involving a demonstration of the home resource that included: a DVD, manual and play cards^b^. During parent-training, the following dimensions were covered: providing feedback before, after and during play-dates, helping children resolve conflicts and strategies that promote social interactions. Treatment fidelity was monitored by the therapist recording parent reported treatment adherence weekly.
		*Therapist*. During the intervention, the first and third author met weekly to review video footage and to discuss the first author’s use of techniques. Three occupational therapy students were trained as clinic assistants to ensure uniformity in the treatment delivery. The students were present across all phases of the intervention for 47% of the 319 sessions. Students ensured the clinic playroom was set up consistently for each session and observed the therapist’s adherence to protocols for: children’s video feedback, playroom interactions, parent-therapist discussions and the completion of checklists.
	Minimise contamination across treatment conditions	Only one session was conducted at a time in the clinic. Participants did not have contact with or the contact details of other participants in the program. Children’s medication status was maintained and parents did not to commence additional therapy during the waitlist/intervention.
Monitor and improve receipt of treatment	Ensure participant comprehension and ability to use target strategies	Children’s comprehension of strategies and content had been developed across three pilot studies^a^. Parent training and ongoing consultation was used to ensure parents’ comprehension and use of material. Parents discussed home DVD content with their child to ensure children comprehended the key messages. Therapist-child discussions in video-feedback sessions ensured children understood self-modelling footage. In week 7, children first played without the therapist to allow the observation of target skills when support was withdrawn. A post-test measurement was taken in week 10.

*Notes*. Framework for process evaluation strategies adapted from: Bellg et al. [41], Borrelli et al. [42], Czajkowski [43] and Spillane et al. [40].

^a^ Wilkes-Gillan et al. [21, 32, 33].

^b^The Ultimate Guide to Making Friends [44].

#### Intervention clinic play sessions

During one hour clinic sessions in weeks 1–3, 5, 7 and 10, the therapist conducted a 20-minute video-feedback session with the children. To promote consistency between the clinic and home, parents also joined the video-feedback session. Children were shown 3-minutes of edited video footage of themselves playing from the previous week. Green slides with a key message appeared before footage of desired social skills (e.g., Great sharing!) and red slides appeared when skills required improvement (e.g., We can listen to our friend). The therapist discussed the footage with the children using key terminology to assist them in identifying positive “green” actions that would make their play more fun (e.g., share ideas). The therapist cued parents and playmates in the conversation (e.g., ‘What do you think made that play so much fun?’). The therapist then supported the children to identify three key actions to remember before entering the playroom (video-feed-forward).

While engaging in mutually enjoyable, cooperative play for 25-minutes, the therapist modelled the desired pro-social skills: sharing, perspective-taking, problem-solving, negotiating and responding to a playmate’s verbal and non-verbal cues. Additional support was provided to help the children negotiate when disagreements occurred. Prompts and key terminology that linked back to the video-feedback session, (e.g., ‘Remember to talk to fix the problem’) were used. The therapist also used gestures and key words to assist children in identifying the emotional states of their playmates, for example, ‘She’s turning away—too rough!’ and to highlight the consequences of their actions, ‘If you play your friend’s game, then they’ll play your game’. Parents observed these sessions through the one-way mirror. The therapist then spent 15-minutes with the parent discussing how the skills and strategies in the session could be implemented at home.

In weeks 7 and 10, children were given video-feedback. Thereafter, they played in the playroom without therapist support for 20-minutes. In week 7, this allowed for the evaluation of children’s social skills when support was withdrawn. The therapist and parent observed the children’s play from behind the one-way-mirror and discussed which social skills would benefit from further support. The therapist then directed parents to the corresponding home-modules addressing these skills to complete in weeks 8 and 9 (see Table 2). In week 10 a post-test measurement was taken.

#### Intervention home-modules

Parents received training in week 1 for one hour on how to deliver home-modules (see Table 2). Module allocation was based on children’s baseline social item ToP scores and ongoing observations. To deliver weekly home-modules, the parent read a manual chapter and watched the DVD episode with their child. Parents engaged their children in a discussion about the fictional characters on the DVD who modelled pro-social behaviours in contexts familiar to children, such as taking turns on equipment at the park. Undesirable responses, (e.g., yelling) were shown, before the characters modelled how to repair the social interaction (e.g., problem-solving). For more information about the DVD see [33]. During weeks 4, 6, 8 and 9, parents facilitated a 40-minute play-date at their home, inviting the playmate involved in the study. Parents used play cards and the terminology learnt during the course of the intervention to give the children feedback before, during and after the play-date. The cards were: green (Great play! Keep going!), red (Let’s stop and think), and purple (3 things to remember).

#### Home follow up

One month after the intervention, the first author visited the homes of children with ADHD to video-record them and their playmate playing. The author spent 10 minutes talking with the children before a 20-minute play session was recorded.

#### Treatment adherence

The first author recorded clinic session attendance. Parents reported on the frequency of viewing the home-modules (DVD and manual use) and facilitating play-dates (see Table 2) [40].

#### Ratings of children’s play

Each child’s pre, post and one-month follow up video-recorded play sessions were randomised and scored by a blinded rater. The rater was an occupational therapist who had been trained and calibrated on the ToP. To be calibrated, raters independently score a set of existing videos, which are compared to hundreds of other raters who have scored the same videos. Using Rasch analysis, it was determined the rater’s scores were reliable as the goodness-of-fit statistics were within the required parameters (*MnSq* < 1.4; standardised value ≤ 2). The rater scored 101 play sessions (see Fig 1).

### Data Analysis

Prior to conducting the main analysis, missing outcome data, blinded ratings of the children’s play and between group differences at baseline were examined. Data analysis was then conducted in five stages: 1) difference in change between the intervention-first and control-first group, 2) overall changes in children’s play skills, 3) changes in social ToP items, 4) predictors of intervention change, and 5) parent reported treatment adherence. Even though raters were blinded to the familial relationships of participants, to account for potential dependence in data with three families enrolling more than one child with ADHD, a sensitivity analysis was conducted to ensure the findings remain robust. Only the first enrolled child in each family was included in the sensitivity analyses (intervention group *n* = 12; waitlist group *n* = 13). The significance or non-significance of the results did not change. Therefore, the below results are reported on the total sample N = 29).

#### Missing outcome data

Two participants dropped out, with both groups having equal missing data (see Fig 1). The two discontinuing participants had completed < 10% of the process and demographic data were incomplete. These cases were excluded from the analysis.

#### Blinded ratings of children’s play sessions

We used the Rasch analysis Winsteps program (version 3.70.1) [45] to convert children’s ToP raw ordinal scores into interval level scores. In this process, an overall measure score was also calculated for each child across each time point. To obtain interval level scores for each participant, ToP raw scores were entered into an existing database containing scores of children with ADHD and typically-developing children (*n* = 406). Goodness-of-fit statistics for people and items were within the parameters set *a priori* (*MnSq* < 1.4; standardised value ≤ 2).

#### Between group differences at baseline

ToP and demographic data were entered into SPSS version 19. The Kolmogorov-Smirnov test indicated that data were normally distributed. Thus, paired samples *t*-tests were used to compare differences between interval level mean ToP scores and CCBRS data of the ADHD (intervention-first vs. control-first group) and playmate (intervention-first vs. control first group) groups at baseline. McNemar’s test was used to compare the difference of paired nominal demographic data (i.e., gender, playmate type) (see Table 1). There was only one statistically significant systematic difference between the playmate groups (more male playmates in the intervention-first group). Thus data from the intervention-first (*n* = 15) and control-first (*n* = 14) groups were combined (*n* = 29) for analyses pertaining to hypotheses 2 and 3 and predictors of intervention change. Data from the groups were combined in order to achieve the sample size determined by the power calculation.

#### Difference in change between intervention and control group

Winsteps-generated person measure scores were entered into IBM SPSS (version 19) to compare mean ToP scores over time. As data were normally distributed, *t*-tests for dependent samples were used to compare the difference in change in overall play skills from pre to post intervention (intervention-first) and from baseline one and two (10 week wait; control-first group). Significance levels were set at *p* < .05. Additionally, a *t*-test for dependent samples was used to compare the change for the control-first group from baseline one and two over the 10-week wait period.

#### Overall changes in children’s social play outcomes

In stage 2 of the analysis, a repeated measures one way ANOVA was conducted to compare overall changes in children’s play skills in peer-to-peer interactions pre, post and one-month following the intervention. Complete data were available for all 29 children and Mauchly’s test indicated the assumption of sphericity had not been violated. Post hoc Fisher’s Least Significance Difference (LSD) tests were used to compare children’s play skills from: 1) pre to post intervention, 2) post intervention to the one-month follow up, and 3) pre intervention to the one-month follow up. All significance levels were set at *p* < .05 and Cohen-*d* effect sizes were calculated by: group (time point mean—time point mean)/pooled SD for group measure scores. Effect sizes were interpreted as: small ≥ .20, medium ≥ .50, or large ≥ .80 [46].

#### Changes in social ToP items scores over time

During stage 3, we examined changes and the effect size in the nine social ToP items: pre, post and one-month following the intervention. Winsteps provides interval level scores for each item that are based on the entire population in the data set. As our sample represents a subset of the full data set, when using scores for individual items for this subset of children, raw scores were used. The ToP overall ordinal scores were strongly correlated to ToP interval level scores across each time point (*Spearmans* ρ = .942–.999, *p* < .001). As raw scores are ordinal level data and were not normally distributed, non-parametric tests were used for analyses using the ToP social items. Friedman test calculations examined changes in each social ToP item mean scores across all time points. Significance was set at *p* < .05.

The *r* effect size [46] was then used to calculate the effect sizes for non-parametric social ToP item data. In this calculation, the effect size (i.e., *r*), is obtained by dividing the Wilcoxon *Z* score by the square root of the sample size (i.e., 29); *r* = *Z* /√ N [47]. Cohen’s guidelines for *r* are: small effect ≥ .1, medium effect ≥ .3 or large effect ≥ .5 [46, 47]. To obtain the Wilcoxon signed rank tests for related samples, ToP social item mean scores were compared from: 1) pre to post, 2) post to follow up, and 3) pre to follow up intervention. A Bonferroni correction was applied to control the false discovery rate associated with multiple testing [48]. Applying this correction, a new familywise significance threshold was set by dividing the overall .05 significance level by the number of Wilcoxon tests performed within each time group comparison (i.e., 9) [48].

#### Predictors of intervention change

Stage 4 involved examining variables that may predict intervention change. Change was calculated by obtaining the difference in ToP scores for each child across each of the time points. Pearson’s correlations were used to determine participant variables (i.e., variables listed in Table 1) that were associated with any point of change. Variables including: the severity of child symptomology and ADHD presentation (as measured by the CCBRS), co-morbid social, behavioural and language difficulties (as measured by the CCBRS), baseline ToP score, medication use, parent occupation and education and variables relating to the invited typically developing playmate (i.e., age, age difference to child with ADHD, gender, if the playmate was a peer or sibling, and CCBRS symptomology) were included because such factors have the potential to influence intervention outcomes. Variables that had a moderate correlation (*r* ≥ .30) with intervention change were included in stepwise linear regression models. Model 1 examined variables that predicted pre to post intervention change. Model 2 examined variables that predicted post intervention to follow up change. Model 3 examined variables that predicted pre intervention to follow up change.

#### Treatment adherence

Stage 5 involved the analysis of parent reported treatment adherence. Mean percentages were calculated for the following factors related to treatment adherence: clinic session attendance, home-modules, play-dates, and overall intervention adherence.

## Results

### Hypothesis 1: Difference in change between intervention and control group

The change in the overall play skills of children with ADHD in the intervention-first group during their intervention phase (pre to post intervention) was significantly greater than the change in the overall play skills of children with ADHD in the control-first group during their 10 week wait period (*t* = 8.02, *p* < .001; 95% CI = 18.79–31.71). The change in the overall ToP scores for the intervention-first group was: 23.8 (mean), 6.1–48.3 (range), 10.6 (SD). The change in the overall ToP scores for the control-first group during their 10-week wait period was: -1.4 (mean), -7.5–10.1 (range), 5.4 (SD). During the waitlist period, children’s baseline 1 mean ToP score was 49.29 (SD = 7.14) and the baseline 2 mean ToP score was 47.90 (SD = 10.30). For the control-first group, no significant differences were found in children’s social play skills over the 10-week period of no intervention (*t* = -.959, *p* = .355; 95% CI = -4.51–1.74).

### Hypothesis 2: Overall Changes in Children’s Play Outcomes

There was a significant main effect of time on the overall ToP measure scores for children with ADHD following the intervention, *F*(2, 27) = 63.2, *p* < .001. Post hoc LSD analysis indicated children’s overall play scores improved significantly from pre to post intervention: mean pre = 46.65 (SD: 11.0), mean post = 67.79 (8.4), *p* < .001, 95% CI = 16.27–26.00, *d* = 1.5. A significant difference was also found from pre intervention to the one-month follow up: mean pre = 46.65 (11.0), mean follow up = 69.68 (7.5), *p* < .001, 95% CI = 16.98–29.08, *d* = 1.6. No difference was found from post intervention to the one-month follow-up: mean post = 67.79 (8.4), mean follow-up = 69.68 (7.5), *p* = .873, 95% CI = -2.59–6.38, *d* = .3.

### Hypothesis 3: Changes in Social ToP Item Scores

There was a significant main effect of time for all social ToP item scores following the intervention. Post hoc analysis indicated social ToP item scores improved significantly from pre to post intervention and from pre intervention to the one-month follow up. No difference was found from post intervention to the one-month follow up (see Table 3).

**Table 3:** Changes in ToP social skill item scores over time.

		Descriptive Statistics						Friedman’s^c^		Post Hoc Pairwise Comparison^d^			
		Pre		Post		Follow up		Pre-post-follow up		Pre to post (*p*)		Pre to follow up	
ToP Item^a^	Brief Item Description	Med	IQR^b^	Med	IQR	Med	IQR	*Χ*^*2*^	*p*	*χ*^*2*^	*p*	*χ*^*2*^	*p*
Initiates	The child’s skill/ability to initiate a new activity with another	1.0	1.0	2.0	1.0	2.0	2.0	15.570	< .001	.897	.002	.655	.038
Negotiates	The child’s skill/ability to negotiate with others using ‘give and take’	1.0	1.0	3.0	1.0	3.0	1.0	34.842	< .001	1.103	< .001	1.069	< .001
Shares	The child’s skill/ability to allow others to use toys or ideas about the game	1.0	1.0	3.0	0.5	3.0	0.0	45.596	< .001	1.328	< .001	1.362	< .001
Supports	The child’s skill of helping others; using verbal support or by physical assistance	1.0	1.0	3.0	1.0	3.0	0.5	42.271	< .001	1.328	< .001	1.362	< .001
Social extent	The extent/proportion of time the child interacts with others	1.0	2.5	3.0	0.0	3.0	0.0	34.413	< .001	.966	< .001	1.000	< .001
Social intensity	The intensity/depth of the child’s interactions with other’s during play	1.0	1.0	3.0	1.0	3.0	0.0	45.512	< .001	1.276	< .001	1.362	< .001
Social skill	The child’s skill/ability to interact with others in cooperative and competitive play	1.0	1.5	3.0	1.0	3.0	1.0	39.474	< .001	1.293	< .001	1.293	< .001
Gives cues	The child’s skill/ability to give verbal and non-verbal cues to others	2.0	1.0	3.0	0.0	3.0	0.5	31.654	< .001	.810	.006	.897	.002
Responds to cues	The child’s skill/ability to respond to others' verbal and non-verbal cues	1.0	1.0	3.0	1.0	3.0	0.5	41.238	< .001	1.207	< .001	1.276	< .001

Notes.

^a^Items can be rated on: skill, extent and intensity (degree).

^b^IQR = Interquartile range.

^c^Friedman’s two-way ANOVA.

^d^Post hoc pairwise comparison tests *p* = adjusted *p*-value after post hoc Dunn-Bonferroni test. Post to follow up: none of the differences were statistically significant.

After the Bonferroni correction (i.e., .05 / 9 = .006), significance was set at *p* < .006. From pre to post intervention, there was a large effect size for the change in all nine social ToP items (*r* = .64–.86). From pre intervention to the one-month follow up, there was a large effect size for the change in eight of the social items (*r* = .70–.84), with a medium effect size for the item *initiates* (*r* = .45). From post intervention to one-month follow up, there was a small effect size for the change in five of the nine social items (*r* = -.11 –.26; see Table 4).

**Table 4:** Effect sizes of ToP social skill item scores.

	Pre to post		Post to follow up		Pre to follow up	
ToP Item	Z	*r*.	*Z*	*r*.	*Z*	*r*.
Initiates	3.46	.64^c^	-0.65	-.11	2.42	.45^b^
Negotiates	4.19	.78^c^	-0.11	-.02	3.99	.74^c^
Shares	4.54	.84^c^	0.88	.16^a^	4.53	.84^c^
Supports	4.63	.86^c^	0.92	.17^a^	4.54	.84^c^
Social extent	3.90	.72^c^	0.45	.08	3.88	.72^c^
Social intensity	4.60	.85^c^	1.31	.24^a^	4.46	.83^c^
Social skill	4.57	.84^c^	0.47	.09	4.36	.80^c^
Gives cues	3.71	.69^c^	1.41	.26^a^	3.79	.70^c^
Responds to cues	4.41	.82^c^	0.90	.17^a^	4.39	.81^c^

*Notes*. The *r* effect size [46] was used to calculate the effect sizes for nonparametric data. In this calculateion, the effect size (i.e., *r*), is obtained by dividing the Wilcoxon *Z* score by the square root of the sample size; *r* = *Z* /√ N [47]. Cohen’s guidelines for *r* are:

^a^small effect ≥ .1,

^b^medium effect ≥ .3 or

^c^large effect ≥ .5 [22, 46]. Bonferroni adjusted *p* values are reported. After the Bonferroni correction, significnace was set at *p* < .006. Pre to post: all items, except for *‘initiates’* were significant *p* < .001. Post to follow up: none of the differences were statistically significant *p* > .05. Pre to follow up: all items, except for *‘initiates’* were significant *p* < .001.

### Predictors of Intervention Change

One variable was correlated with children’s pre to post intervention change and was therefore entered into the Model 1 regression analysis: children’s pre intervention/baseline ToP score (*r* = -.693, *p* < .001) (see Table 5). Three variables were correlated to post intervention to follow up change and were entered into the Model 2 regression: age difference in the child dyad (*r* = .464, *p* < .01), if the sibling was a playmate (*r* = -.403, *p* < .032), and children’s post intervention ToP score (*r* = -.650, *p* < .001) (see Table 5). Two variables were correlated to pre intervention to follow up change and were entered into the Model 3 regression: if the sibling was a playmate (*r* = .413, *p* < .026), and children’s pre intervention ToP score (*r* = -.810, *p* < .001) (see Table 5).

**Table 5:** Final models of linear regression analyses: Predictors of intervention change.

Included Variables	*β*	*p*	CI^a^
Model 1: Pre to post change			
Baseline ToP score^b^	-.650	< .001	-.917 to -.383
Model 2: Post to follow up change			
Post-test ToP score^c^	-.739	< .001	-1.079 to -.398
Model 3: Pre to follow up change			
Baseline ToP score^d^	-.946	< .001	-1.216 to -.676

*Notes*. ToP = Test of Playfulness [31].

^a^CI = 95% confidence interval for *β*.

^b^A higher pre intervention baseline ToP score negatively predicted greater pre to post intervention change.

^c^A higher post intervention score negatively predicted greater change from post intervention to the one-month follow up.

^d^A higher pre intervention baseline ToP score negatively predicted greater change from pre intervention to the one-month follow up. Excluded variables that were entered into the models are listed in section 3.3.

The results of Model 1 indicated a higher pre intervention baseline ToP score predicted lower pre to post intervention change. The results of Model 2 indicated a higher post intervention score predicted lower change from post intervention to the one-month follow up. The results of Model 3 demonstrated a higher pre intervention baseline ToP score predicted lower change from pre intervention to the one-month follow up. Participants’ demographic information and other variables (i.e., symptom severity in children and parent education and occupation reported in Table 1) did not predict intervention change or the effectiveness of the intervention.

### Parent-Reported Treatment Adherence

All families reported completing ≥80% of the intervention. Mean percentages for adherence to the clinic, home-module and play-date components are reported in Table 6.

**Table 6:** Parent reported treatment adherence.

Intervention component	Mean %	Range %	Reasons for non-attendance/adherence
Clinic session attendance (Weeks: 1–3, 5, 7, 10)	98.3	88–100%	4 families missed one clinic session, reasons: playmate sick, playmate overseas (x 2), change to family schedule.
Home-modules: DVD and manual (Weeks: 1 or 2, 3–10)	89.1	66–100%	6 families missed two modules (77%), 1 family missed three modules (66%), reasons: busy family schedule.
Play-date with the playmate Weeks: 4, 6, 8 & 9	87.1	75–100%	15 families missed one play-date (77%), reasons: changes to playmate’s schedule.
Overall intervention adherence	92.1	80–100%	-

*Notes*. Treatment adherence was based on parent-report and was recorded on a weekly basis by the therapist.

## Discussion

We evaluated the effectiveness of a play-based intervention designed to improve the social play skills of children with ADHD. This study adds to the evidence-base of psycho-social interventions using play as a mode of intervention delivery by examining its effectiveness with a larger sample size using a strong methodological design [32, 34]. This study also addresses an urgently needed area of research—to establish effective interventions for improving the social difficulties of children with ADHD in multiple contexts [23, 24].

The between group change was significantly higher in the intervention-first group (pre to post intervention) compared to the change in the control-first group (10-week wait period). The control-first group did not change during the control period of no intervention. The effect of the “waiting” list control condition should be considered. Parents allocated to a waitlist control may have an expectation that they are to wait to change until receiving the intervention; which could enhance the lack of change seen in the control-first group over their 10-week wait period [49].

When evaluating the effect once all children had received the intervention, the intervention yielded a large treatment effect (*d* = 1.5 and 1.6 respectively) for improving the play skills of all children with ADHD in peer-to-peer interactions. Our findings support that the change was due to the intervention rather than external factors. The social play skills of children allocated to the control-first group did not improve over the 10-week no-treatment period and the groups were not significantly different in any of the key variables upon entry to the study. Further, regression analyses demonstrated potential confounding variables (e.g., medication use, child symptomology, parent occupation and education) did not predict intervention change (see Table 1). These results are promising as improving the social difficulties of children with ADHD has remained an elusive goal for most psychosocial interventions [23, 24].

Lower baseline ToP scores predicted greater intervention change. This is a promising finding as it demonstrates that children with severe social skills deficits benefited most from the intervention. Moreover the finding indicates that the intervention format is developmentally appropriate in addressing the underlying social skills deficits. We suspect multiple components within the intervention catered to children’s individual developmental needs and led to improvements in their social play skills, regardless of their skills upon entry to the program.

Similar to our previous findings [21, 32, 33], children in this study were motivated and seemed to enjoy watching themselves in the video-feedback sessions. With therapist support, the children were able to identify reasons for discontinued cooperative play (e.g., ‘He said, “Stop” and I didn’t listen’) and identify social skills needed for future interactions (e.g., ‘We need to listen to our friend’). These observations are consistent with children’s improvement on ToP social items, *gives* and *responds to cues*. Our findings are consistent with previous research that found children with social difficulties benefit from video-modelling techniques when they are used to exemplify the discussion and identify social skills (Shukla-Mehta, Miller, & Callahan, 2010).

We found, that including typically-developing playmates was both motivating and beneficial for children with ADHD. Like previous research, we found typically-developing peers from the target child’s natural social environments were key to modelling desired social skills and facilitating cooperative play [21, 32, 33, 50, 51]. In fact, we postulate that the playmates served as an important change agent as they became adept at handling challenging social behaviours. Playmates remembered the cognitive target skills and strategies from the video feedback session and applied them while playing in the playroom, acting as in-the-moment reminders for children with ADHD (e.g., ‘Remember to share ideas about the game—it’s more fun!). These findings are reflected in children’s improvement on social ToP items: *extent of time spent in social interaction*, *social skill* (i.e., cooperative play), and *shares*. Consistent with previous research, we found the intervention also supported the development of the playmates’ social skills [21, 32, 33, 51]. As the intervention progressed, playmates’ were increasingly able to negotiate to have their own needs met, while supporting children with ADHD (e.g., ‘I want to play a different game’ or ‘we’ll play 10-minutes of each person’s game’).

As expected with school-aged children, on occasion, the playmates did not engage in pro-social behaviour [52]. This provided opportunities for the therapist to encourage children with ADHD to *negotiate* and take on the perspective of their playmates’ by *supporting* their needs (e.g., ‘Let’s play your friend’s game for 5 more minutes. They are having fun’). These situations were emphasised during the video-feedback session the following week and encouraged children to pre-emptively *support* their playmate’ needs above their own (e.g., ‘We’ll play your game first this week, that will make it green play’); a known difficulty for children with ADHD [11–13, 32].

We suspect the components within the intervention were effective as they supported the core difficulties of children with ADHD as social interactions unfolded [13, 15]. Using video-modelling, the therapist supported children to pre-empt the social skills they would need during social play interactions and to anticipate the impact of their actions on their playmates emotional state. In the playroom, the therapist and playmate supported children with ADHD to problem-solve when difficulties unfolded during spontaneous child-led/initiated play interactions; helping them realise such situations were within their control [9, 13]. Key to engaging children in the intervention and presenting them with opportunities to develop their social skills and positive interactions was the therapist ensuring children were engaged in activities they themselves devised and determined to be play. A key determinant of whether or not a child regards an activity as play, extending from a positive affective interaction is a child’s playfulness; their disposition or tendency to play [29, 53, 54]. In this instance, a child’s playfulness is most commonly defined and observed by the characteristics that comprise it, being, 1) intrinsic motivation (the child engages in the activity because they enjoy the process, rather than for external reward), 2) internal control (the child has some control over their actions, directions and outcomes of the activity), 3) freedom from the constraints of reality (the child chooses how close to objective reality their play is) and, 4) the framing of play (the child’s ability to give and read social cues about how to interact, cues that “we’re playing”) [29, 53, 54].

While the intervention required weekly activity commitments from parents, parents’ reported excellent treatment adherence (≥ 80%), demonstrating that this commitment was feasible. We found the level of time commitment from parents enhanced children’s treatment outcomes, with parents playing a key role in assisting children to generalise skills to the home environment. While children’s outcomes did not improve significantly from post intervention to the one-month follow up, treatment gains increased slightly (*d* = .30) and were maintained (pre intervention to follow up, *d* = 1.6). These findings are consistent with previous research, finding increased parent involvement strengthened treatment outcomes [21, 32, 33, 55–57]. As anticipated through the use of a manualised parent component (that aligns with the social skills targeted in the intervention), parents reported using the strategies to assist their child in generalising the skills to settings beyond the home environment (e.g., at birthday parties, on holidays and at school). These reports are consistent with parents’ semi-structured interview responses from the pilot parent-delivered intervention [58]. However, parents’ perceptions of implementing the intervention still require formal investigation. This is particularly important as children with ADHD have difficulty with skill generalisation [25] and experience social difficulties across multiple social contexts [59].

Anecdotally, parents expressed that through ongoing supported social opportunities (i.e., play-dates and clinic play sessions), the children’s interactions deepened and friendships developed, a finding supported by improvements on the ToP item *intensity of social interaction*. However, these findings require formal investigation.

### Limitations and Implications for Future Research

The relatively small sample size and use of convenience sampling may limit the generalisation of the findings to the broader population of children with ADHD. Data from children with ADHD from the same family could not be pooled into one observation per family due to the small sample size and only three families enrolling more than one child with ADHD. As such, we were unable to conduct a multilevel analysis to account for family clustering. To address potential dependence in the data we conducted a sensitivity analysis, whereby only the first enrolled child from each family was included in the analysis to ensure our findings remained robust [60]. Additionally, the severity of neuropsychological impairments in children and the presence of ADHD symptomology in parents was not collected, which could have impacted treatment adherence and the effectiveness of the intervention. It is likely that the improvement of children’s social play skills were a result of the play-based intervention. However, we cannot rule out the possible influence of demand characteristics. It is possible that parents formed an interpretation of the trials purpose and changed their behaviour and/or parent report responses to fit those expectations. However, this is unlikely to have influenced the behaviour and skill performance of children with ADHD to the same extent due to their known difficulties with social cognition and behavioural inhibition [9, 13, 14, 17].

As playmates and parents were key components of the intervention, further research is required to evaluate the social play outcomes of the playmates involved in the study and parents’ perceptions of the intervention [61]. Longer-term research is needed to establish if treatment gains are maintained across the home and clinic contexts and if parents continue to use the strategies after the intervention, in multiple contexts [20, 25]. While parent occupation and education, indicators of socioeconomic status (SES), did not impact the results of this study, it is possible that families with low SES may have difficulty meeting the demands of this intervention. Such families may benefit from an intervention design that is weighted towards more therapist supported clinic sessions. Further research is needed to examine the impact of the parent characteristics on the optimal mode of intervention delivery (i.e. greater therapist supported clinic sessions vs greater home-based activity). While we sought to improve the social skills of children with ADHD, it is possible that other changes occurred as a result. Future studies on the intervention could consider examining secondary outcomes that may occur as a result from the intervention/improvement in social skills (i.e., cognitive functioning, communication skills). Another important avenue for future research would be to evaluate the potential benefits of natural childhood play in reducing ADHD symptoms. In alignment with the guidelines for developing complex interventions, other avenues for further research could include adapting and trialling the intervention: 1) with a broader range of developmental disabilities (e.g., autism spectrum disorders), 2) for implementation in schools, and 3) ensuring therapists can implement the intervention in community settings [27, 33].

## Conclusions

The results from this study demonstrated the play-based intervention was effective for improving the social play skills of children with ADHD aged 5 to 11 years in peer-to-peer interactions in the clinic and home environments. These findings support interventions using the context of play and a child treatment component with parent- and peer-mediated components when aiming to improve the social difficulties of children with ADHD.

### Trial Information

The trial was registered with the Australian New Zealand Clinical Trials Registry (registration number: ACTRN12614000973617). The trial protocol can be accessed online at, http://www.ANZCTR.org.au/ACTRN12614000973617.aspx.

## Supporting Information

S1 File CONSORT Checklist. (PDF)

S2 File Registered Trial Protocol. (PDF)

S3 File Trial Protocol in Ethics Application. (PDF)
