# Supplementary material for: A Randomised Controlled Trial of a Play-Based Intervention to Improve the Social Play Skills of Children with Attention Deficit Hyperactivity Disorder (ADHD)
Source: PLoS One. 2016 Aug 16;11(8):e0160558. doi: 10.1371/journal.pone.0160558 (PMC4987013; doi:10.1371/journal.pone.0160558)
Supplement: S2 File — (PDF) [file pone.0160558.s002.pdf]

ANZCTR
Australian New Zealand Clinical Trials Registry

Questions in **bold** text are mandatory. **(*)**

**Request Number:**	367030
**Current Page:**	Review

Trial from ANZCTR

**Trial ID**	ACTRN12614000973617
**Trial Status:**	Registered
**Date Submitted:**	2/09/2014
**Date Registered:**	10/09/2014
	**Retrospectively registered**

Page 1

**Public title**	A play-based intervention to improve the social play skills of children with Attention Deficit Hyperactivity Disorder (ADHD)
**Study title in 'Participant-Intervention-Comparator- Outcome (PICO)' format**	The effect of a play-based intervention on social play skills of children with Attention Deficit Hyperactivity Disorder.
**Secondary ID [1]**	Nil known
UTN	U1111-1161-0904
Trial acronym	

Page 2

**Health condition(s) or problem(s) studied:**	
Social difficulties	
Attention Deficit Hyperactivity Disorder (ADHD)	
**Condition category:**	**Condition code:**
Mental Health	Other mental health disorders

Page 3

**Descriptions of intervention(s) / exposure**	Children will receive a 10-week play-based intervention called 'The Ultimate Guide to Making Friends'. After attending a baseline assessment, children and their invited typically-developing playmate will attend the intervention which involves 6 weekly clinic sessions led by an occupational therapist (weeks: 1, 2, 3, 5, 7, 10). All clinic sessions will last 1 hour and involve a video-feedback discussion and clinic play session with the therapist. The child and their playmate will first view video footage of themselves playing from the previous week. The footage will have explicit short clips of footage where the children played well together and where they were not engaged in cooperative play. Children will be helped by the therapist to discuss the footage and brain storm three 'green skills' while they play in the playroom together (e.g., share toys, play my friend's game, talk to fix problems). The therapist and children then went into the playroom where the therapist modelled desired social skills while playing with the children and facilitated cooperative play between them.<br><br>During weeks 4, 6, 8 & 9 children with ADHD will complete home activity facilitated by their parents which includes a DVD and manual resource and weekly play-date with the playmate they invited to the study. Parents will first read the prescribed manual chapter - each containing information about a social skill (e.g., perspective-taking) (15 minutes) before watching the correlating DVD episode involving fictional characters with their child (30 minutes). The DVD will cue parents when to pause the episode and have a discussion with their child about what they just watched. The DVD was designed to give children ideas about play with friends and how to repair social interactions when a problem occurred. During the week parents also invited the playmate from the study over for a 40 minute play-date. Before the playmate arrived parents where encouraged to talk with their child and come up with three things to remember while they played with their friend (based on what they watched from the DVD episode).<br><br>Parent-reported treatment adherence was recorded by therapists on a weekly basis across the following categories: clinic sessions, DVD and manual activity and play-date.<br><br>A post-test measurement will be taken in the week 10 clinic session. A 1 hour follow-up will then be conducted at the home of children with ADHD one month after the intervention. This involves a video-taped session of the child and their playmate playing in the home environment and a semi-

	structured interview completed by parents.
**Intervention Code:**	Behaviour
**Comparator / control treatment**	After all children with ADHD complete a blinded baseline assessment they be randomly allocated in pairs to an 1) intervention (immediate start) or 2) no-treatment 10-week waitlist/control group.<br><br>After the 10-week wait period, children will commence the intervention.
**Control group**	Active

Page 4

**Primary Outcome:**	To investigate if the social play skills of children with ADHD change following the intervention (As measured by the Test of Playfulness, ToP; Bundy, 2004)
Timepoint:	Pre- to post-intervention (10 weeks) and from pre-intervention to the 1-month home follow-up
**Primary Outcome:**	To investigate if the pragmatic language abilities of children with ADHD change following the intervention (As measured by the Pragmatics Observational Measure, POM; Cordier et al. 2014)
Timepoint:	Pre - to post-intervention (10 weeks) and from pre- intervention to the 1-month home follow-up
**Secondary Outcome:**	To investigate if children with ADHD demonstrate changes in their pragmatic (social use of) language skills as scored by parents using the Children's Communication Checklist 2nd edition; subscales 1) use of context, 2) nonverbal communication, 3) social relations and 4) interests (CCC-2; Bishop 2003).
Timepoint:	Pre- to post-intervention
**Secondary Outcome:**	To investigate changes in the social play (ToP) and pragmatic language skills (POM) of typically-developing playmates of children with ADHD following the intervention
Timepoint:	Pre- to post-intervention (10 weeks) and pre-intervention to the 1-month home follow-up

Page 5

**Key inclusion criteria**	Children with ADHD: had to have a formal diagnosis of ADHD (made by a paediatrician or psychiatrist using recognised diagnostic procedures e.g. DSM-IV). Children continued prescribed medication for ADHD. Parents were asked not to change their child's medication during the study. One parent/primary caregiver also needed to available to participate in the intervention delivery.<br><br>Typically-Developing Playmates: did not have a diagnosis of ADHD and no concerns were raised by parents or teachers about the children's development.<br><br>The Conners Comprehensive Behavior Rating Scales (CCBRS; Conners, 2008) was used as a screening measure to confirm the presence or absence of ADHD symptoms. Children with ADHD needed to score above the clinical cut off (T-Score >70) and playmates needed to score below the borderline clinical cut off (T-score <65).
**Minimum age**	5 Years
**Maximum age**	12 Years
**Gender**	Both males and females
**Healthy volunteers?**	No
**Key exclusion criteria**	The study commenced at the time of the DSM-IV. Children with ADHD were excluded if they were diagnosed with other major neurodevelopmental or psychiatric disorders (e.g. intellectual disability)as well as ADHD.<br><br>Playmates were excluded if they scored above the clinical cut off on the CCBRS.

Page 6

**Study type**	Interventional
**Purpose of the study**	Treatment
**Allocation to intervention**	Randomised controlled trial
Describe the procedure for enrolling a subject and allocating the treatment (allocation concealment procedures)	Before recruitment commenced, an even number of opaque envelopes were prepared and sealed containing a piece of paper with either 'group 1 intervention' or 'group 2 10-week wait'.<br><br>When interested parents phoned the researcher about the study, a baseline assessment was scheduled. Information collected over the phone included the parents/child's name and date/time of the initial baseline assessment (where researchers and participants were blinded to group allocation).
Describe the methods used to generate the sequence in which subjects will be randomised (sequence generation)	A pairwise randomisation method was used. Once two parents had booked in a baseline assessment, a sealed envelope from each pile (group 1 or group 2) was taken to a staff member not involved in the research. A piece of paper containing the two date/times of the baseline assessments was also taken to the staff member. The staff member was asked to use simple randomisation procedures e.g. coin toss to pick one of the two dates/times written on the paper. They were then instructed to shuffle the two envelopes and place this time on one of the sealed envelopes. The researcher left the room while the staff member completed the procedure.

	The envelopes were then placed in a client folder which was taken by researcher into the baseline assessment. At the end of the baseline assessment, the researcher handed the parent the sealed envelope from the client folder (participants and the research/therapist were only blinded for the baseline assessment).
Masking / blinding	Blinded (masking used)
Who is / are masked / blinded (choose all that apply)	The people assessing the outcomes
Assignment	Parallel
Other design features	
Type of endpoint (s)	Efficacy
Statistical Methods/Analysis	Based on 3 previous pilot studies and power/sample size calculations, we determined the sample size needed was 15 children with ADHD per group.<br><br>To calculate sample size we used the G Power calculator and input the: 1) desired power (e.g., 0.8), 2) statistical test (e.g., ANOVA), 3) alpha value (0.05) and 4) expected effect (>.5 large); which generated the minimum sample needed n = 30.<br><br>Anticipated statistical analysis includes: One-way repeated measures ANOVA with post-hoc LSD and Cohen's'-d effect sizes to determine the efficacy of the intervention, and Stepwise linear regression model analysis to determine variables that predict intervention change.

Page 7

Phase	Not Applicable
**Anticipated date of first participant enrolment**	1/05/2013
Date of first participant enrolment	1/06/2013
Anticipated date last participant recruited/enrolled	29/03/2014
Actual date last participant recruited/enrolled	7/06/2014
**Target sample size**	34
Actual sample size	
**Recruitment status**	Closed: follow-up continuing

Recruitment in Australia

**Recruitment state(s)**	NSW
**Postcode:**	2141 - Lidcombe

Recruitment outside Australia

Page 8

**Funding Source:**	Charities/Societies/Foundations
**Name:**	The Rotary Club of Mosman
**Address:**	P.O. Box 88<br>Mosman<br>NSW<br>Australia<br>2088
**Country:**	Australia
**Primary Sponsor**	University
**Name:**	The University of Sydney
**Address:**	Faculty of Health Sciences<br>75 East St<br>Lidcombe<br>NSW<br>Australia<br>2141
**Country:**	Australia
**Secondary Sponsor:**	None
**Name:**	
**Address:**	
**Country:**	

**Has the study received approval from at least one Ethics Committee?**	Yes
Ethics Committee name:	The University of Sydney Human Research Ethics Committee
Address:	Level 6, Jane Foss Russell<br>The University of Sydney<br>NSW 2006 Australia
Country:	Australia
Approval Date:	21/03/2013
Submitted Date:	29/11/2012
HREC:	2013/109
Brief summary	The purpose of the 10-week play-based intervention is to improve the social and communication skills of children with ADHD. We aim to do this by using: the context of play, parents, children's typically-developing playmates and therapist supported clinic sessions.<br><br>We suspect, that both children with ADHD and their playmates will demonstrate significant improvements in their social and communication skills following the intervention and that these skills will also be observed in the home environment.<br><br>We suspect parents may also find the intervention a supportive approach that assists them to develop their child's social and communication skills which are needed for friendships.
Trial website	
Trial related presentations / publications	
Public Notes	
Attachments [1]	http://www.anzctr.org.au/AnzctrAttachments/367030-Appendix G Intervention structure.doc

Principal Investigator

Title:	Ms
Name:	Sarah Wilkes-Gillan
Address:	The University of Sydney, Faculty of Health Sciences, 75 East St Lidcombe NSW Australia 2141
Country:	Australia
Tel:	+61 402402359
Fax:	
Email:	swil8454@uni.sydney.edu.au

Contact person for public queries

Title:	Ms
Name:	Sarah Wilkes-Gillan (PhD Candidate)
Address:	The University of Sydney, Faculty of Health Sciences, 75 East St Lidcombe NSW Australia 2141
Country:	Australia
Tel:	+61 402402359
Fax:	
Email:	swil8454@uni.sydney.edu.au

Contact person for scientific queries

Title:	Ms
Name:	Sarah Wilkes-Gillan
Address:	The University of Sydney, Faculty of Health Sciences, 75 East St Lidcombe NSW Australia 2141
Country:	Australia
Tel:	+61 402402359
Fax:	
Email:	swil8454@uni.sydney.edu.au

Contact person responsible for updating information

Title:	Ms
Name:	Sarah Wilkes-Gillan
Address:	The University of Sydney, Faculty of Health Sciences, 75 East St Lidcombe NSW Australia

Country:	
Tel:	+61 402402359
Fax:	
Email:	swil8454@uni.sydney.edu.au
