# Supplementary material for: A Randomised Controlled Trial of a Play-Based Intervention to Improve the Social Play Skills of Children with Attention Deficit Hyperactivity Disorder (ADHD)
Source: PLoS One. 2016 Aug 16;11(8):e0160558. doi: 10.1371/journal.pone.0160558 (PMC4987013; doi:10.1371/journal.pone.0160558)
Supplement: S3 File — (PDF) [file pone.0160558.s003.pdf]

This section is obligatory

9.1 Describe the project using lay terms wherever possible, including the aims, hypotheses, research plan
and potential significance. Where relevant, provide the projected number, sex, and age range of participants (including inclusion/exclusion criteria). You must satisfy the HREC that the study is scientifically valid (include at least four (4) research references) and conducted in accordance with the accepted ethical principles governing research involving humans.
The description must be no longer than 2 pages and must be in a font size of at least 10 points.

Aim of the study: To evaluate the effectiveness of an intervention for improving the social play skills and pragmatic language of children with ADHD.

Participants:

The proposed study will comprise 50 children with ADHD who will each be paired with a self-selected, typically-developing playmate. The parent/carer of the child with ADHD also will actively participate in the study.
Inclusion and exclusion criteria. All participating children will be between 5 and 11 years of age; both children and parents must be fluent in English (i.e., home/school language being English). ***Children with ADHD.*** Formal diagnosis of ADHD made by a psychiatrist or paediatrician using recognised diagnostic methods such as the American Psychiatric Association's Diagnostic and Statistical Manual 4th edition (DSM-IV; American Psychiatric Association, 2000); scores above the clinical cut-off scores of the ADHD subscales on the Conners Comprehensive Behavior Rating Scale-Parent (CCBRS-P; Conners, 2008). Children will be **included** even if they have conditions commonly occurring with ADHD (e.g., language disorders, learning disorders and oppositional defiant disorder) provided ADHD is the primary diagnosis. Children will be **excluded** if they have other major developmental or psychiatric disorders (e.g., autism, intellectual disability, cerebral palsy). Children will continue using any medication prescribed for ADHD throughout the intervention. ***Playmates.*** Each of the children with ADHD will invite a regular (pre-known) typically-developing playmate of a similar age to participate in the intervention. Because many children with ADHD do not have regular playmates, they may invite a typically developing sibling of a similar age. For the purpose of this study, "typically-developing" children are defined as children who (a) do not have ADHD (i.e., do not meet DSM-IV criteria and have scores below the clinical cut-off on all subscales of the CCBRS-P) and (b) do not have any evidence of other developmental or behavioural difficulties as described by their parents and teachers. ***Parents of children with ADHD*****:** At least one parent/primary carer will be involved in the intervention. Where possible, both parents will be included.

Research plan:

Allocation to groups. As recruitment is likely to be somewhat sporadic, children will be allocated in pairs after they enter the program to either a control or intervention group. To minimize bias, randomisation will be concealed from the investigators involved (Domanski & McKinlay, 2009). A person not involved in the study will hand the investigator an envelope determining group membership; the concealed envelope will be given to the family *following* the initial assessment/testing. The control group will be offered the intervention immediately after a 10-week waiting period.

Format for the Intervention. The 10-week study assesses the effectiveness of a play-based intervention. The intervention is a combination of both the therapist-delivered and parent-delivered versions we tested earlier (Wilkes, et al, 2011; Wilkes et al, in preparation). The combined intervention emphasises therapist-led clinic activity in combination with active parent involvement with therapist support and training. The therapist-led clinic component involves the therapist playing in the playroom with the children in order to model and promote pro-social interactions during the natural context of play. The therapist will begin each clinic session by showing the children video footage of themselves playing from a previous week and planning for the session in the clinic. The parent-delivered component will supplement the clinic-based intervention. The parent-based intervention component is contained within a

DVD and manual and consists of 12 modules. The therapist will support parents over the phone during the weeks they deliver the intervention at home.

Intervention structure. The intervention adopts the following structure (see Appendix G):
Initial assessment visit: Clinic based – All families will enter the program by attending an initial assessment session at the clinic. During this session, all children will complete their baseline play assessment. While supervising the children through a one-way mirror, an initial consultation with the parent will occur. Once the baseline assessment is finished, parents will be given time to complete the associated forms/questionnaires (see Appendix J). During this time the researcher/therapist will provide supervision and keep the children engaged. The role of the therapist during this time is to strictly build rapport and offer companionship to the children while their parents complete forms for the study. To ensure the results of the control group are not influenced, the therapist will not engage in or use any of the strategies/techniques embedded in the intervention. At the end of the session, together, the therapist and parents will open a concealed envelope to find out their group membership. The group membership will have been unknown to both participants and the therapist/researcher until this point.

Week 1: Clinic based – Children's play session with therapist & parent training.
Week 2 & 3: Clinic based - Therapist modelling to demonstrate techniques used in the DVD; video feedback with children; individual feedback from baseline data and strategies discussed with parents
Week 4: Home based - Cover various topics contained in DVD and Manual; phone consultation with therapist
Week 5: Clinic based – see weeks 2 & 3 above
Week 6: Home based – see week 4 above
Week 7: Clinic based – see weeks 2 & 3 above
Weeks 8 & 9: Home based – see week 4 above
Week 10: Clinic based - Post test assessment and feedback

1 Month Follow-up. As part of the follow-up, a semi-structured interview will be conducted by an investigator who was not involved in the intervention delivery process. Interviews will be recorded and conducted over the phone (see Appendix B; Interview topics). Previous parents reported this to be the most convenient method. Additionally, the investigator who assumed the role of therapist will visit the home of the participants to record a free-play session will to assess generalisation of intervention effects to the home environment using the ToP (see Appendix A; Safety protocol).

Outcome measures: (see Appendix J 1-6)

**Primary Measures** (Pre- and post-test as well as 1 month follow-up)	*Test of Playfulness (ToP;* Bundy, 2004). The ToP is a 30-item observer rated instrument suitable for children between 6 months and 18 years. The ToP measures the concept of playfulness as a reflection of the combined presence of four elements: internal control, freedom from unnecessary constraints of reality, intrinsic motivation, and ability to give and read social cues. The ToP is administered in an environment that is supportive of play. It has evidence for good to excellent internal and external reliability and internal and external validity (Bundy, Nelson, Metzger, & Bingaman, 2001) (Appendix J1).
	Pragmatic Protocol (PP; Prutting & Kirchner, 1987*)*. The PP is an observational measure of pragmatic aspects of language such as verbal, nonverbal and paralinguistic aspects of language and based on direct observation. Inter-rater reliability = 0.94 (Appendix J2).
	Structured, Multidimentional Assessment Profiles (S-MAP; Wiig, Larson, & Olson, 2004). The S-MAP measures language and communication abilities across academic, personal, and social contexts. Inter-rater reliability = 0.71-0.88; Intra-rater reliability = 0.86-0.93 (Appendix J3).
**Secondary Measure** (Pre-test and post-test)	*Parent Relationship Questionnaire (PRQ;* Kamphaus & Reynolds, 2008). The PRQ is a parent completed questionnaire designed to assess parenting relationship variables deemed to have a significant impact on a child's social and emotional development. The PRQ-CA (child and adolescent) form consists of 7 subscales and is standardised for parents/guardians of children aged 6 to 18 years. Reliability coefficients for test- retest and internal consistency ranged from .72 to .81. Moderate to high construct validity reports were identified (Rubinic & Schwickrath 2010; Appendix J4).

	The Children's Communication Checklist-2nd edition (CCC-2; Bishop, 2003). The CCC-2 is a caregiver questionnaire that screens for communication problems in children aged 4-16 years of age. It is a valid and reliable tool that includes several aspects of communication including language form (e.g., syntax) and language use (e.g., pragmatic aspects of language such as nonverbal communication and social relations) (Appendix J5).
**Screening Measure**	*Conners Comprehensive Behavior Rating Scale-Parent (CCBRS-P;* Conners, 2008). The CCBRS-P is a comprehensive assessment tool which assesses a wide range of behavioural, emotional, social, academic concerns, and disorders in children and adolescents (ages 6 – 18 years old) including ADHD. Reliability: 0.80 or higher; Concurrent Validity: 0.70 or higher (Appendix J6).

The ToP, PP and S-MAP will be scored by raters blinded to purpose of the study from the video-recorded sessions.

Evaluation:

Outcome evaluation. The effectiveness of the intervention will be measured by comparing the mean pre-test and post-test ToP, S-MAP and PP scores for individuals within the intervention groups using *ANOVA*. Cohen-*d* values will also be calculated. Cohen suggested that an effect size of .20 is small, .50 is medium, and .80 is large. Inferential statistics will be used to analyse the data from secondary assessment sources. A qualitative thematic analysis will be conducted using the data from the semi-structured interviews (Strauss & Corbin, 1990). We expect very few drop-outs. Any parents who discontinued treatment will be invited to participate in the interview.

Potential Significance: Children with ADHD and their families know how hard it is to live with ADHD. This innovative intervention study directly addresses the one thing that children with ADHD themselves have identified as most needed: the ability to make and keep friends. We address the social play and communication skills deficits in children with ADHD by uniquely targeting what is increasingly deemed to be at the root of their social skills problem: lack of interpersonal empathy (Barkley, 2006; Braaten & Rosen, 2000; Cordier, et al., 2009; Marton, Wiener, Rogers, Moore, & Tannock, 2008).
